# Noninvasive assessment of the anaerobic threshold in endurance athletes: an evidence-based narrative review of methods, validity, and field feasibility

**DOI:** 10.1093/bmb/ldag037

**Published:** 2026-10-14

**Authors:** Nicola Maffulli, Johnny Padulo, Francesco Oliva

**Affiliations:** Department of Trauma and Orthopaedic Surgery, Faculty of Medicine and Psychology, University of Rome “La Sapienza”, 00185 Rome, Italy; Centre for Sports and Exercise Medicine, Barts and the London School of Medicine and Dentistry, Queen Mary University of London, E1 4DG London, United Kingdom; School of Pharmacy and Bioengineering, Faculty of Medicine, Keele University, Stoke-on-Trent, ST4 7QB, United Kingdom; Department of Biomedical Sciences for Health, Università degli Studi di Milano, 20133 Milan, Italy; Department of Sports Traumatology, Università Telematica San Raffaele, Via di Val Cannuta, 247, Roma, Italy

**Keywords:** anaerobic threshold, lactate threshold, endurance athletes, heart rate variability, near-infrared spectroscopy, critical power, wearable technology

## Abstract

**Introduction or Background:**

The anaerobic threshold (AnT) marks the transition between heavy and severe exercise intensity domains and guides training prescription and performance monitoring. Criterion methods (blood lactate and gas exchange) are accurate but invasive, costly, or impractical outside laboratories, prompting the search for noninvasive surrogates. This review evaluates the validity of these estimation procedures rather than the underlying physiology.

**Sources of Data:**

MEDLINE (via PubMed) was searched from inception without language restrictions, supplemented by reference-list and citation searching. Eligible studies compared noninvasive AnT indices, including heart rate variability (HRV; including detrended fluctuation analysis [DFA] α1), cardiac repolarization instability, heart rate deflection/field slope-variation methods, critical power (CP)/ critical speed (CS), near-infrared spectroscopy (NIRS), the talk test, and machine-learning (ML) models, with criterion standards. Risk of bias was assessed using QUADAS-2. Eighty-eight reports were synthesized narratively; no meta-analysis was performed.

**Areas of Agreement:**

DFA α1, NIRS breakpoints, and CP/CS show strong associations with laboratory criteria and increasing field applicability through wearables. Accuracy and feasibility trade-offs require method selection according to the threshold, sport, and required precision.

**Areas of Controversy:**

Correlation indicates group-level association rather than individual agreement; Bland–Altman and reliability analyses are more informative for substitution. Criterion standards are not interchangeable, and heart rate deflection is not consistently identifiable. Evidence in female athletes remains limited.

**Growing Points:**

Multimodal wearables integrating HRV, muscle oxygenation, and running power with ML may improve field-based threshold estimation.

**Areas for Developing Research:**

Priorities include standardized reporting, transparent bias assessment, female-athlete validation, and broader systematic and meta-analytic synthesis.

## Introduction

The anaerobic threshold (AnT) is among the most extensively studied constructs in exercise physiology, yet it remains one of the most contested [1]. Conceptually, it denotes the highest exercise intensity at which the appearance of lactate in the blood is matched by its clearance, and above which a sustained metabolic steady state cannot be maintained [1]. As it delimits the transition between the heavy and severe intensity domains, the AnT and its aerobic counterpart are central to calibration of endurance training zones, individualization of exercise prescription, and monitoring of performance [2]. A plethora of operational definitions has accrued, including the lactate threshold (LT), the onset of blood lactate accumulation (OBLA), the individual AnT, the ventilatory thresholds (VT), and the respiratory compensation point (RCP), each carrying characteristic and predictable measurement error [1–3].

Two families of laboratory measurement dominate criterion-standard determination [4,5]. Gas exchange analysis identifies the VT, and the V-slope method detects the disproportionate rise in carbon dioxide output that accompanies bicarbonate buffering of lactic acid [4]. Blood sampling identifies LTs and, through repeated constant-load trials, the maximal lactate steady state (MLSS), widely regarded as a definitive index of the maximal metabolic steady state [5]. The critical power (CP) construct, derived from the hyperbolic power-duration relationship, has been advanced as an alternative criterion for the boundary of sustainable exercise [6]. These reference indices are related but not interchangeable, and their proximity varies with protocol and exercise modality [7].

The impetus to determine the AnT without repeated venepuncture or expensive metabolic carts is longstanding [8]. Early laboratory work sought noninvasive surrogates from ventilatory and heart rate responses [8], and the deflection from linearity in the running speed to heart rate relationship was proposed to identify the AnT in the field [9]. Field applications of the speed to heart rate relationship were subsequently validated in middle and long distance runners and in master athletes, with strong correlations between the slope variation point and directly measured thresholds [10–12]. The physiological responses that accompany attainment of the AnT, spanning ventilation, cardiovascular function, catecholamines, heart rate and electromyographic activity, provide numerous candidate signals for noninvasive detection [13].

Over the past decade, wearable sensors, smartphone applications and computational methods have widened the range of candidate surrogates. The short-term scaling exponent of detrended fluctuation analysis (DFA) of HRV (DFA α1) has attracted particular attention as an index that changes continuously with intensity and can demarcate thresholds without prior calibration [14]. A recent systematic review with meta-analysis reported trivial differences and very strong correlations between HRV-derived thresholds and VT or LTs [15], and a separate meta-analysis examined the proximity of CP to metabolic and VTs [7]. However, these syntheses each address a single modality family. No review has compared the full spectrum of noninvasive methods, from autonomic and muscle-oxygenation signals to field performance models, within a common framework of criterion-standard agreement and field feasibility.

Accordingly, the present evidence-based narrative review was undertaken to identify, appraise and compare noninvasive methods for AnT assessment in endurance athletes. Further aims were to summarize their agreement with criterion standards, and to map their practical feasibility, so as to guide method selection for coaches, clinicians and sports scientists. This review addresses the validity of testing methods and surrogate indices for estimating the AnT and its aerobic counterpart, rather than the underlying physiological phenomena themselves. The validity of a given index as an estimation procedure does not by itself constitute evidence for or against the physiological construct it targets. The need is underscored by continuing efforts to derive simple, field-deployable estimates of the LT and peak oxygen uptake from submaximal and anthropometric variables [16].

## Methods

### Protocol and registration

This is an evidence-based narrative review. To support transparency and reproducibility, the evidence was identified through a documented, structured literature search rather than an exhaustive systematic-review protocol, and the flow of identification and selection is reported in Fig. 1. A structured search, explicit eligibility criteria and formal risk-of-bias appraisal were incorporated to increase transparency and reduce selection bias; the narrative, rather than systematic-review, label was retained because the review was not prospectively registered, dual independent screening was not applied at every stage, and the marked heterogeneity of indices, protocols and criterion standards across the included literature precluded meta-analytic pooling.

**Figure 1:**
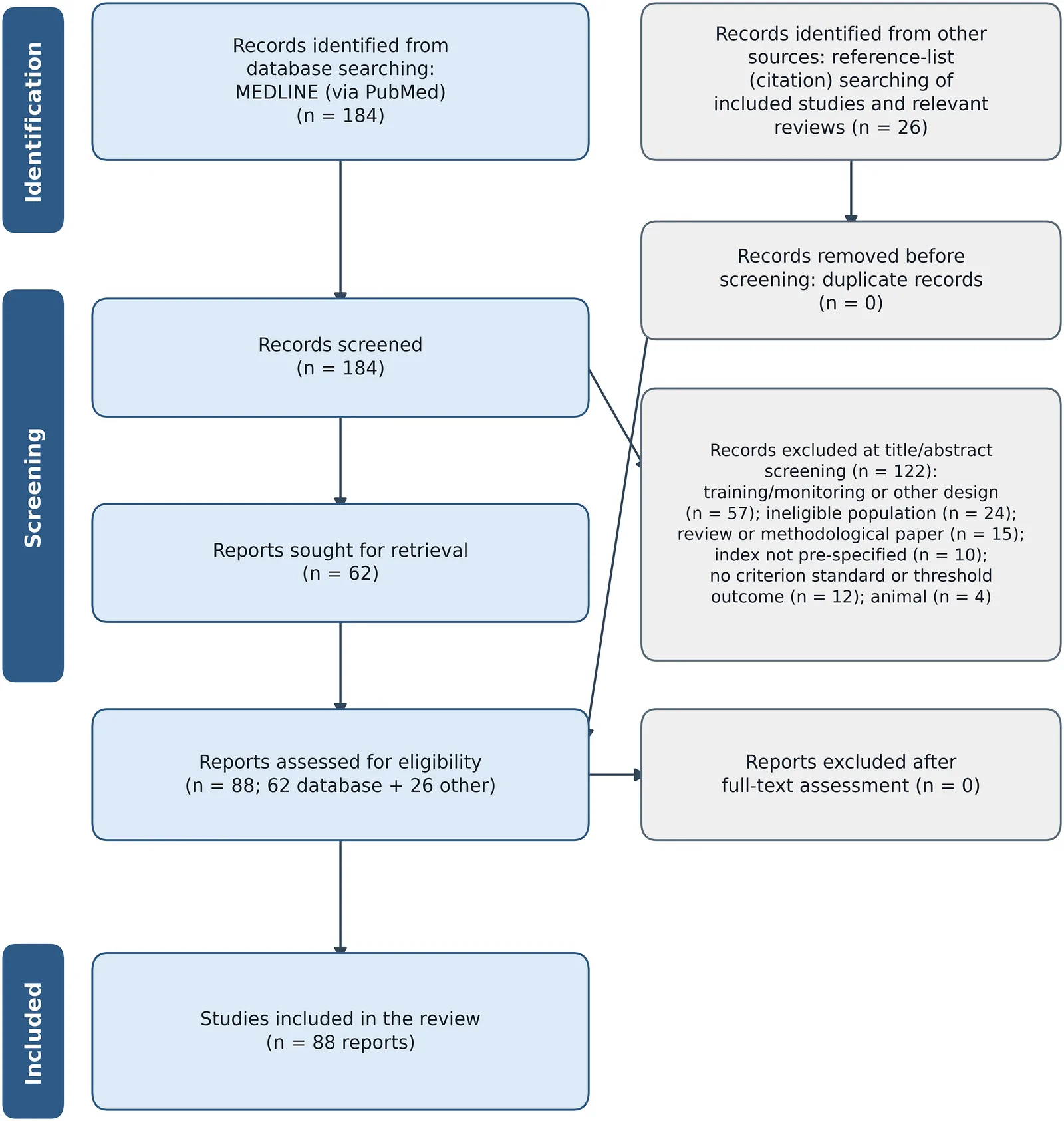
Flow of literature identification and selection.

### Eligibility criteria

Studies were eligible if they enrolled endurance-trained or competitive athletes (runners, cyclists, triathletes, rowers, or cross-country skiers) of any sex; evaluated at least one noninvasive index of the AnT or its aerobic counterpart; compared the index against a criterion standard; and reported agreement, correlation or diagnostic accuracy outcomes. Studies addressing the aerobic threshold (AerT) were retained alongside those addressing the AnT because the two transitions are frequently reported within the same validation study and because several indices, notably DFA α1 and near-infrared spectroscopy, are validated against both boundaries using a shared protocol; findings specific to the AerT are identified as such throughout. Reviews, editorials, conference abstracts without extractable data, and studies confined to clinical or sedentary populations were excluded, although mixed cohorts were retained when athlete data could be isolated. Where studies enrolled team-sport or clinical participants alongside endurance athletes, only data from endurance athletes are synthesized in the main narrative and such studies are identified as providing supporting rather than primary evidence. The full eligibility framework is presented in Table 1.

**Table 1:** Eligibility criteria (PICOS framework).

**Parameters**	**Inclusion**	**Exclusion**
Population	Endurance-trained or competitive athletes (runners, cyclists, triathletes, rowers, cross-country skiers), any sex, adolescent to masters age	Sedentary or clinical populations only, unless athlete subgroup data are separable
Index test	Noninvasive index of the anaerobic or aerobic threshold: HRV/DFA α1, heart rate deflection point, critical power or critical speed, NIRS muscle oxygenation breakpoint, ECG cardiac repolarization instability (dT°), talk test, machine-learning or regression model	Invasive-only assessment; index not intended to estimate a metabolic threshold
Comparator	Criterion standard: blood lactate threshold or OBLA, MLSS, gas exchange or ventilatory thresholds, respiratory compensation point, or critical power	No criterion-standard comparator reported
Outcomes	Agreement (Bland–Altman bias and limits of agreement), correlation, standardized mean difference, sensitivity or reproducibility	No quantitative agreement or accuracy outcome
Study design	Cross-sectional validation, cohort or randomized studies with extractable data	Narrative reviews, editorials, abstracts without extractable data

### Information sources and search strategy

MEDLINE (via PubMed) was searched from inception, without language restriction, using a combination of controlled vocabulary and free-text terms for the AnT, noninvasive assessment modalities and endurance athletes. In addition, the reference lists of all included studies and of relevant systematic reviews were screened, and forward citation searching of key papers was performed. The search strategy is reported in Table 2. Database searches were conducted through 30 June 2026. Reliance on a single database is acknowledged as a limitation of the search strategy (see Limitations); the inclusion of Scopus, Web of Science, Embase, or SPORTDiscus would extend coverage, but the present strategy prioritized transparency of a single, fully reported database search (Table 2) over exhaustive multi-database retrieval, consistent with the evidence-based narrative, rather than systematic, aim of the review.

**Table 2:** Search strategy and supplementary searching.

**Source**	**Search strategy/terms**
MEDLINE (via PubMed)	(‘anaerobic threshold’ OR ‘lactate threshold’ OR ‘ventilatory threshold’ OR ‘maximal lactate steady state’ OR ‘critical power’ OR ‘critical speed’) AND (‘heart rate variability’ OR ‘detrended fluctuation’ OR Conconi OR ‘heart rate deflection’ OR ‘near-infrared spectroscopy’ OR ‘cardiac repolarization’ OR ‘talk test’ OR ‘machine learning’ OR wearable) AND (athlete^*^ OR runner^*^ OR cyclist^*^ OR triathlete^*^ OR endurance). Searched from inception, without language restriction; 184 records retrieved. The strategy uses 19 Boolean operators, within the PubMed limit.
Reference-list and citation searching	Reference lists of all included studies and relevant systematic reviews screened; forward citation tracking of key papers

### Study selection and data extraction

Titles and abstracts of all records were screened against the eligibility criteria independently by all three authors (N.M., F.O. and J.P.), with disagreements resolved by consensus discussion. Data were extracted on population, sport, exercise modality and protocol, index method and analytical procedure, criterion standard, and agreement outcomes (correlation coefficients, Bland–Altman bias and limits of agreement, standardized mean differences, sensitivity and reproducibility). Data extraction followed the same independent, three-author procedure.

### Risk of bias

Methodological quality and risk of bias were assessed with QUADAS-2, tailored to the review question across the domains of participant selection, index test, reference standard, and flow and timing [17]. Across the included literature, risk of bias was generally low for the participant-selection and reference-standard domains, reflecting the athlete-only eligibility criteria and the use of established criterion methods, but was more frequently unclear or high for the index-test domain, reflecting variability in recording hardware, artefact-correction procedures and incremental-protocol design across studies. The domain-level QUADAS-2 judgements for each modality family, across the four risk-of-bias and three applicability domains, are summarized in Table 3.

**Table 3:** Summary of QUADAS-2 methodological quality and risk of bias, by modality family.

**Method family**	**Risk of bias**				**Applicability concerns**		
	**Participant selection**	**Index test**	**Reference standard**	**Flow and timing**	**Participant selection**	**Index test**	**Reference standard**
HRV/DFA α1	Low	Unclear	Low	Low	Low	Low	Low
ECG repolarization instability (dT°)	Unclear	Unclear	Low	Low	Low	Unclear	Low
HR deflection/field slope-variation (Conconi and related)	Low	High	Low	Unclear	Low	Low	Low
Critical power/critical speed	Low	Unclear	Low	Unclear	Low	Unclear	Unclear
NIRS (SmO₂) breakpoint	Low	High	Low	Low	Low	Low	Low
Talk test	Low	High	Low	Low	Low	Low	Low
ML/regression models	Unclear	High	Low	Unclear	Low	Unclear	Low

Ratings denote the predominant domain-level QUADAS-2 judgement across the studies within each modality family. Participant selection, index test and reference standard are appraised for both risk of bias and applicability; flow and timing is appraised for risk of bias only.

Cell shading: green, low; amber, unclear; red, high risk of bias or applicability concern. Risk of bias was assessed with QUADAS-2 [17].

Abbreviations: AnT, anaerobic threshold; DFA, detrended fluctuation analysis; dT°, beat-to-beat vector change of cardiac repolarization; ECG, electrocardiographic; HR, heart rate; HRV, heart rate variability; LT, lactate threshold; ML, machine learning; NIRS, near-infrared spectroscopy; RCP, respiratory compensation point; SmO₂, muscle oxygen saturation; VT, ventilatory threshold.

### Synthesis

Given the heterogeneity in indices, protocols and criterion standards, the evidence was synthesized narratively and grouped by modality family; no meta-analysis was undertaken. For each modality family the review summarizes the direction and consistency of agreement with criterion standards and the practicality of field deployment. The flow of literature identification and selection is summarized in Fig. 1. Because many included studies report correlation coefficients as their primary or sole agreement statistic, an important synthesis caveat applies throughout this review. Correlation quantifies the strength of a linear association across a sample, and can remain high even when individual-level differences are large and practically unacceptable. Bland–Altman bias and limits of agreement, typical error, the intraclass correlation coefficient, and the standard error and minimal detectable change are considerably more informative when the objective is to determine whether a noninvasive index can substitute for a criterion method in a given athlete. Throughout the sections: Results and Discussion, the term agreement is therefore reserved for studies reporting such individual-level statistics, whereas correlation is reported as such, and is not treated as evidence of interchangeability.

## Results

### Literature identification

The results of searching, screening and eligibility assessment are summarized in Fig. 1. The MEDLINE (via PubMed) search returned 184 records. Title and abstract screen excluded 122 records: 57 as training, monitoring or other ineligible designs; 24 for an ineligible population; 15 as reviews or methodological papers; 10 for an index test outside the pre-specified families; 12 for the absence of a criterion standard or threshold outcome; and 4 as animal studies. The remaining 62 reports were eligible; a further 26 reports were located through reference-list and citation searching, giving 88 reports assessed for eligibility. The synthesis below is organized by modality family; the principal methods, the threshold each targets and representative validity evidence are collated in Table 4, and their positioning against the exercise intensity domains and along axes of feasibility and validity is depicted in Fig. 2.

**Table 4:** Summary of noninvasive methods for anaerobic threshold assessment.

**Methods**	**Principle/index**	**Target threshold**	**Representative validity evidence**	**Field feasibility**	**Key refs**
HRV/DFA α1	Short-term fractal scaling exponent of RR intervals; declines with intensity	AerT (α1 = 0.75); AnT (α1 = 0.5)	Close agreement of HR at α1 = 0.5 with VT2; meta-analysis of 50 studies, *r* = 0.85; validated in elite cyclists and triathletes; device and artefact bias possible	High (wearable, smartphone, real-time)	14–15, 19–36
ECG repolarization instability (dT°)	Beat-to-beat vector change of cardiac repolarization from high-resolution ECG	AnT (≈ LT2/VT2)	Maximal dT°-HR discordance correlated with LT (*r* = 0.84 in adults; *r* = 0.96–0.98 in team-sport athletes); independent VT2 estimation	Low to moderate (high-resolution multi-lead ECG)	37–39
HR deflection/field slope-variation point (Conconi and related)	Departure from linearity, or slope-variation point, of the HR to work-rate (or speed) relationship	AnT (near LT2)	Predicts LT when deflection identifiable; field slope-variation point correlates with lactate, ventilatory and OBLA thresholds in young and master runners (*r* ~ 0.82–0.90); not always identifiable; degraded by glycogen depletion	High (HR monitor only)	8–9, 11–12, 40–66
Critical power/critical speed	Asymptote of power/speed to time-to-exhaustion relationship	AnT (heavy/severe boundary)	Group-level agreement with MLSS and turnpoint but wide individual limits; may represent MMSS more closely than MLSS in runners	Moderate (multiple trials, no blood)	6–7, 67–74
NIRS (SmO₂) breakpoint	Muscle oxygen saturation breakpoints during incremental exercise	AerT (first) and AnT (second)	Breakpoint not different from LT2 in cyclists, strong r; desaturation rate tracks lactate; site- and device-dependent	Moderate to high (portable sensor)	75–86
Talk test	Ability to speak comfortably at a given intensity	Around VT1 (positive) to VT2 (negative)	First difficulty approximates VT; sometimes closer to LT; consistent across modalities; coarse resolution	Very high (no equipment)	87–93
ML/regression models	Prediction of threshold velocity/intensity from submaximal and somatic inputs	AnT/RCP	Better prediction of AnT than AerT; reproducible in large cohort; limited external validation	High (once model deployed)	94–96

**Figure 2:**
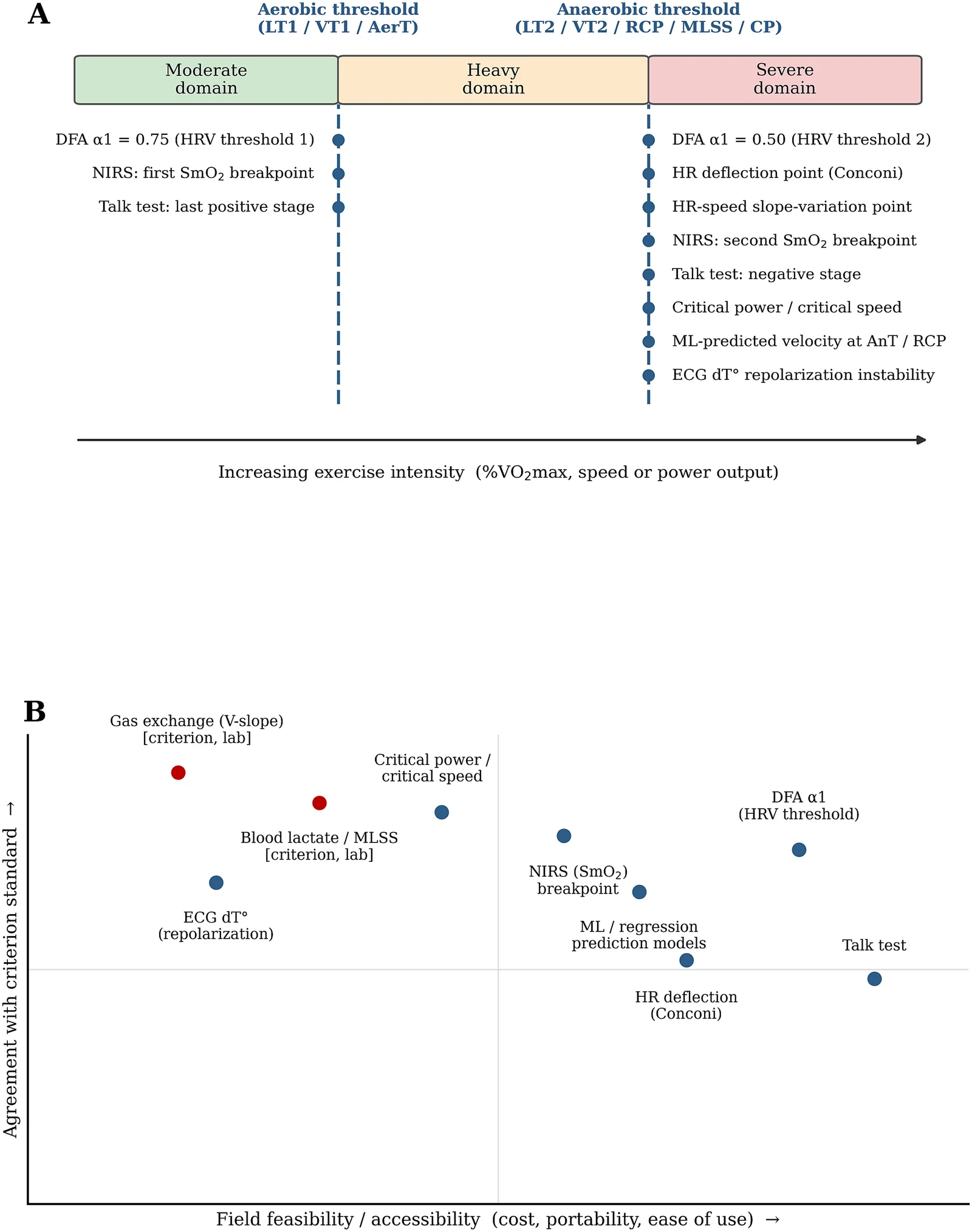
Conceptual synthesis of noninvasive methods for anaerobic threshold assessment. (A) Positioning of each noninvasive index against the exercise intensity domains and the aerobic and anaerobic threshold boundaries. (B) Qualitative mapping of methods along axes of field feasibility (cost, portability and ease of use) and agreement with the criterion standard; the laboratory criterion standards (gas exchange and blood lactate or MLSS) are shown in red for reference. Positions are illustrative, reflect only the qualitative direction and consistency of evidence in the cited studies, and are not derived from a formal, predefined scoring or ranking system or from pooled quantitative estimates; the panel should not be interpreted as a quantitative ranking of method validity.

### Reference standards and terminology

Because noninvasive indices are validated against different criterion standards, their reported accuracy is partly a function of the comparator chosen. The principal criterion standards and threshold terms are defined in Table 5. Two physiological events recur across the literature: a first metabolic transition, variously the first LT or first VT, corresponding to the AerT; and a second transition, the second LT, second VT, RCP, MLSS or CP, corresponding to the AnT [1,2,4–7]. These reference standards are not interchangeable: CP and critical speed (CS) in particular are derived from a distinct methodological principle, the power or speed to time-to-exhaustion relationship, rather than from blood or gas exchange kinetics, and should be regarded as a related but methodologically independent boundary rather than as directly equivalent to LT2, VT2 or MLSS (Table 5). The MLSS is laborious to determine, requiring several constant-load visits, and we showed that the mean of the two VT provides a practical steady-state approximation that reduces the number of trials required [18].

**Table 5:** Criterion standards and threshold terminology.

**Term (abbreviation)**	**Definition**	**Boundary/domain**
First lactate threshold (LT1)/aerobic threshold	First sustained rise in blood lactate above baseline during incremental exercise [2]	Moderate to heavy
Second lactate threshold (LT2)/anaerobic threshold	Highest intensity at which lactate production and clearance remain in equilibrium [1,2]	Heavy to severe
Ventilatory thresholds (VT1, VT2)	Gas exchange breakpoints; VT2 approximates the respiratory compensation point [3]	Moderate/heavy and heavy/severe
RCP	Point of hyperventilation relative to carbon dioxide output above VT2 [3]	Heavy to severe
OBLA	Fixed blood lactate reference (commonly 4 mmol/L) [2]	Heavy to severe
MLSS	Highest constant load at which blood lactate remains stable over time [4]	Heavy/severe boundary
CP/CS	Asymptote of the power (or speed) to time-to-exhaustion relationship; proposed criterion boundary [5,6]	Heavy/severe boundary
Gas exchange (V-slope)	Regression method detecting excess carbon dioxide output from bicarbonate buffering [3]	Reference for VT/AnT

### HRV and DFA α1

HRV indices, and in particular the short-term DFA scaling exponent DFA α1, have emerged as the most intensively studied noninvasive surrogates. DFA α1 declines progressively with increasing intensity, from correlated (fractal) towards uncorrelated (random) beat dynamics. A DFA α1 value of 0.75 has been associated with the AerT or first VT [19], and a value of 0.5, signifying loss of fractal correlation, with the second VT or AnT [14]. In an incremental treadmill study, the heart rate at a DFA α1 of 0.5 agreed closely with that at the second VT [14,20]. A subsequent update consolidated the AerT association across recreational, elite and clinical populations [21]. Agreement has been reproduced during prolonged constant-load running [22], and extended to patients with chronic heart failure, in whom the second HRV threshold tracked the second VT although the first was less reliable [23]. Sport-specific validation supports the approach in trained endurance athletes: in elite cyclists, the first HRV threshold agreed closely with the first LT, with high correlations for power output. In elite triathletes the DFA α1 threshold matched the first LT, although agreement for the second threshold was weaker in both elite cyclists and elite triathletes [24,25]. The most robust overall evidence derives from a systematic review with meta-analysis of 50 studies and 1160 participants, which reported trivial standardized mean differences and very strong correlations between HRV-derived and reference thresholds (*r* = 0.85 for both the first and second thresholds) [15].

Practical caveats are important: artefact correction and recording device type introduce bias, negligible below ~3% missed beats but discernible thereafter, together with a systematic offset between electrocardiographic (ECG) and photoplethysmographic recordings [26,27]. Nonetheless, validated open-source and smartphone implementations now permit real-time field determination [28], and everyday workout data have been used to relate cycling power to DFA α1 for intensity-zone monitoring [29].

This update adds substantial concordance evidence: in trained and sub-elite cyclists and in recreational runners, DFA α1 values of 0.75 and 0.5 tracked the VT and LTs, agreement being generally closer for the second than for the first threshold, with a chest-belt DFA α1 of 0.75 corresponding to the first VT only in a nonfatigued state, and with wide individual limits of agreement that temper individual prescription [30–34]. Time- and frequency-domain HRV indices likewise approximated the AnT during discontinuous cycling and estimated the second VT during ski-mountaineering field testing [35,36].

### Cardiac repolarization instability (ECG)

A distinct autonomic approach analyses beat-to-beat instability of cardiac repolarization from a high-resolution electrocardiogram. The three-dimensional beat-to-beat vector change of repolarization (dT°) rises with intensity in parallel with heart rate, then shows an abrupt transient discordance; the point of maximal discordance between dT° and heart rate defines a repolarization-based threshold. In healthy adults, this threshold correlated strongly with the LT (*r* = 0.84 and *r* = 0.82 for two lactate-threshold methods) [37]. In a prospective validation in 65 professional and amateur team-sport athletes, the repolarization-based threshold correlated very strongly with the LT (*r* = 0.96 and *r* = 0.98) [38]. An independent group, analysing the temporal profile of a ventricular repolarization instability index and its low-frequency oscillations, estimated the second VT noninvasively, with most estimates falling within one power increment of the reference [39]. The method is highly accurate but presently requires a high-resolution multi-lead electrocardiogram and specialized signal processing, which currently limits its field feasibility.

### Heart rate deflection point and field slope-variation methods

The heart rate deflection point, the departure from linearity in the heart rate to work-rate relationship during incremental exercise, was the original field-based noninvasive method [9]. A relationship exists between the deflection point and the second lactate turnpoint, but its validity for detecting the AnT is uncertain, the deflection is not always identifiable, and results depend heavily on protocol [40]. A modified Conconi protocol predicted the LT in distance runners in whom a deflection could be identified, with steady-state confirmation, but a deflection was absent in a proportion of athletes [41]. Agreement is further degraded by physiological perturbation: under glycogen depletion, the heart rate threshold ceased to track the LT [42]. The method is therefore highly accessible, but of variable and conditional validity.

The heart rate deflection concept also underpins a family of field methods based on the slope-variation point of the running speed to heart rate relationship. In young middle distance runners, the slope-variation point determined noninvasively, both in the laboratory and in a track field test, correlated strongly with the directly measured lactate, ventilatory and blood lactate accumulation thresholds (*r* generally 0.82–0.90), the closest agreement being between running speed at the field slope-variation point and at the laboratory LT; these noninvasive indices occurred at a percentage of maximal oxygen uptake comparable with that of adult endurance runners [11]. A companion laboratory method for noninvasive threshold determination [8] and validation in master endurance runners [12] support the approach across the age range, and independent work in long distance runners and cyclists likewise reported significant agreement between a noninvasive field test and VT [43]. As with the Conconi test, however, the slope-variation point is not identifiable in every athlete, which constrains individual application. When a deflection is present, its intensity approximates the ventilatory or respiratory compensation threshold in trained cyclists, rowers, runners and professional team-sport athletes [44–51], and a modified fast-ramp Conconi protocol reproduces the deflection heart rate in runners [52]. Against blood lactate criteria, however, agreement is inconsistent: several studies in cyclists, runners and rowers found that the deflection or updated Conconi test overestimated or failed to predict the LT, or was not reproducible [53–57]. Other studies reported acceptable agreement with lactate, ventilatory or MLSS references and with performance [58–63]. The founding Conconi field studies established the coincidence of the speed to heart rate deflection with the lactate AnT across running, swimming and several other endurance disciplines [64–66].

### CP and CS

CP and its running analogue CS are derived from the relationship between power or speed and time to exhaustion across a small number of constant-load trials, and require no blood sampling or gas analysis. CP has been proposed as the criterion boundary of the maximal metabolic steady state [6]. In runners, critical velocity, MLSS velocity and lactate turnpoint velocity did not differ significantly at the group level, but the limits of agreement were too wide to permit accurate estimation of one from another in individuals [67]. CP derived from time-to-exhaustion trials and from the three-minute all-out test both exceeded MLSS power, and the three-minute test showed wide limits of agreement [68]. In runners, CS did not differ from the intermittent MLSS and correlated strongly with it [69], and evidence that oxygen uptake can stabilize at speeds slightly above MLSS suggests that CS may represent the maximal metabolic steady state more closely than MLSS [70]. A meta-analysis confirmed significant but imperfect correlations between CP and MLSS, the VT and the RCP, with systematic overestimation or underestimation depending on the comparator [7]. CP and CS thus offer physiologically grounded, field-deployable estimates of the AnT with good but not exact agreement. Wearable running-power technology has extended the concept to the field: the Stryd foot-pod CP corresponded most closely to the second VT or the OBLA and predicted performance [71,72]. A step-ramp-step protocol estimated the maximal metabolic steady state accurately from running power though not from speed [73], and the 9/3-minute running critical-power test located CP at essentially the same intensity as the RCP in high-calibre athletes [74].

### Near-infrared spectroscopy

NIRS quantifies muscle oxygen saturation (SmO_2_), and breakpoints in the oxygenation signal during incremental exercise coincide with metabolic thresholds. In elite cyclists, the SmO_2_ breakpoint measured with a wearable device did not differ from the second LT in power or heart rate, and correlated strongly with it [75]. The rate of muscle desaturation was closely correlated with blood lactate across intensities, and breakpoints coincided with the first and second LTs [76]. Measurement site matters, however: breakpoints in the vastus lateralis predicted systemic aerobic-capacity indices better and occurred earlier than those in the gastrocnemius [77]. Device validation supports the reliability of portable monitors [78], but findings are not uniformly positive: using one wearable device, SmO_2_ thresholds alone did not accurately predict LT power, which was recovered only through proprietary processing [79]. Additional validation reinforces this pattern while underscoring its conditionality. A portable NIRS device estimated the second VT in well-trained runners [80], and the SmO_2_ zero-slope power was determined with excellent reliability against the maximal metabolic steady state [81]. In earlier work, the muscle oxygenation breakpoint approximated the MLSS in distance runners and triathletes [82], and a wearable near-infrared device predicted the LT in runners [83]. Agreement is not uniform, however. Deoxyhaemoglobin breakpoints agreed with the second LT in slope-based but not speed-based running protocols [84], agreement with the VT was only weak to moderate in recreational endurance and CrossFit athletes [85]. In an incremental cycling comparison, near-infrared, HRV and VT coincided at the group level but differed markedly within individuals [86]. NIRS therefore provides accurate, portable and continuous threshold estimation, subject to sensor placement and device processing.

### Talk test

The talk test infers threshold intensity from the ability to speak comfortably during exercise. Comfortable speech is generally possible below the VT or LT and becomes difficult above it. The test is consistent across walking, jogging, cycling and other modalities [87]. Primary validation shows that the point at which speech first becomes difficult approximates the VT across treadmill and cycle exercise [88], although in some cohorts the talk-test stages align more closely with the lactate than the VT [89]. Consistent with this, the negative stage of the talk test approximated the ventilatory or respiratory compensation thresholds in highly trained and well-trained cyclists [90,91], aligned with those thresholds when combined with a percentage of maximal running speed in competitive athletes [92], and produced training intensities appropriate for well-trained individuals when used to guide prescription [93]. The talk test is inexpensive and equipment-free, and valid for prescribing moderate to vigorous intensity, but its resolution is coarse and it is less suited to precise or high-intensity prescription.

### Machine-learning and regression prediction models

Computational models estimate threshold intensities from submaximal, physiological and anthropometric inputs. Ensemble machine-learning (ML) models estimated aerobic and AnT from cardiopulmonary exercise data, with better prediction of the anaerobic than the AerT [94]. Regression and ML models derived from a large athlete cohort identified oxygen uptake, blood lactate, pulmonary ventilation and somatic variables as the strongest predictors of velocity at the AnT and RCP, with reproducible accuracy [95]. This model incorporates blood lactate among its input variables: it is therefore more accurately classified as a hybrid rather than a strictly noninvasive predictive approach. Models restricted to noninvasive cardiopulmonary and anthropometric inputs, and algorithms embedded in consumer wearable devices, are considered separately below. Consumer wearable algorithms extend model-based estimation to the field, though with a systematic offset: a widely used running watch underestimated the lactate-threshold pace relative to a standardized blood lactate field test, while estimating threshold heart rate more closely [96]. Such models can personalize prescription, but depend on the quality and representativeness of their training data: external validation in independent athlete cohorts remains limited.

## Discussion

LT2, VT2, RCP, MLSS and CP/CS reflect related but distinct physiological constructs derived from different methodological principles (Table 5). Therefore, the agreement reported between a noninvasive index and any single criterion standard should be interpreted specifically against that comparator rather than generalized across the family of AnT-related thresholds. This review reports the landscape of noninvasive AnT assessment across seven modality families, and integrates them within a common framework of criterion-standard agreement and field feasibility (Fig. 2, Table 4). Two themes emerge. First, several methods now achieve strong correlation, and, in some instances, satisfactory individual-level agreement, with laboratory criterion standards, notably DFA α1, NIRS and CP or CS. Others, particularly the heart rate slope variation/deflection point, show modest and conditional validity. Second, accuracy and feasibility are frequently inversely related: the most accessible methods are seldom the most precise, and method choice entails a deliberate trade-off. ECG repolarization instability exemplifies this tension: it achieves very high correlation with the LT, including in team-sport athletes, but requires laboratory-grade recording. Individual-level agreement statistics for this method remain, however, sparsely reported [37,38].

Method selection should be guided by the threshold of interest, the sport and the precision required. For demarcation of the AerT and low-intensity training zones, DFA α1 at 0.75 and the first NIRS breakpoint are supported, including in trained cyclists and triathletes [24,25]. For the AnT itself, DFA α1 at 0.5, the second NIRS breakpoint, CP and CS, and validated prediction models offer the strongest evidence. Sport-specific and biomechanical factors modulate performance: signal quality in cycling differs from running, upper-body involvement and movement artefact affect autonomic and oxygenation signals, and sensor site and device processing influence NIRS [26,77,79,97].

In practical terms, DFA α1 and the talk test are best suited to field monitoring and recreational or self-guided training given their minimal equipment requirements. NIRS and CP or CS offer a reasonable balance of accuracy and feasibility for club-level or subelite athletes able to undertake a short familiarization or multi-trial protocol. ECG repolarization instability or laboratory gas exchange remain preferable where individual-level precision is paramount, as in elite athletes testing or clinical exercise prescription.

The validity attributed to any surrogate is partly an artefact of the comparator and protocol. It is also partly an artefact of the statistic reported: correlation coefficients, which dominate this literature, describe group-level association and should not be read as evidence that a method can substitute for its criterion standard in an individual athlete, for which Bland–Altman or reliability-based agreement statistics are required. Because criterion standards themselves diverge, notably CP, MLSS and the VT, apparent agreement or disagreement must be interpreted against the specific reference used [7,67,68,70]. Recording hardware, artefact-correction settings and incremental protocol design materially affect autonomic indices [26], and the nonidentifiability of the heart rate deflection point in some athletes limits this approach [40,41]. A recurring pattern across studies is that correlation with the criterion standard tends to be stronger for the first than for the second threshold, which would have direct implications for severe-domain prescription. However, this pattern has not been consistently confirmed across populations, exercise modalities and protocols, and warrants formal comparison rather than assumption. Standardized reporting of device, protocol and analytical parameters is therefore essential.

Beyond athletic populations, noninvasive threshold assessment has clear translational value, for example in cardiac rehabilitation, where HRV thresholds have been validated against VT in chronic heart failure [23]. Extension of the approach beyond running and cycling, for example to anaerobic-endurance monitoring in competitive swimming, further illustrates its cross-disciplinary reach [98]. Continued development of simple predictive equations for the LT and peak oxygen uptake from submaximal tests extends accessibility to recreational and clinical settings [16]. Such noninvasively determined thresholds have long served as a practical tool for prescribing exercise intensity in applied physiological research, for example when setting a treadmill workload relative to the running speed at the AnT [10]. Integration of autonomic markers with power or pace, rather than reliance on any single signal, is a promising direction for durability and fatigue monitoring [21].

Looking forward, the convergence of autonomic, muscle-oxygenation and mechanical-power signals within a single wearable platform, interpreted through ML or artificial-intelligence-based algorithms, represents a promising direction for noninvasive threshold estimation [99,100]. Such multimodal approaches may reduce dependence on any single signal and its specific artefacts, provided that model outputs are validated against individual-level agreement statistics and tested prospectively in independent athlete cohorts rather than relying on correlation with the training sample alone.

This narrative review has limitations. Heterogeneity in indices, protocols, criterion standards and athlete calibre constrains quantitative pooling and may limit generalizability. The literature search relied on a single database (MEDLINE via PubMed), supplemented by citation and reference-list screening; although this strategy captured the great majority of relevant validation studies through forward and backward citation tracking, it may have missed literature indexed exclusively in Scopus, Web of Science, Embase or SPORTDiscus. The predominance of small validation studies conducted in trained men is a further important limitation. Most validation studies to date have enrolled few or no female endurance athletes, and broad generalization of the reported accuracy to female athletes is not currently supported. Dedicated validation in female endurance-athlete cohorts, accounting for potential sex-related differences in autonomic and metabolic responses to incremental exercise, should be prioritized. The possibility of publication bias favouring positive validation findings has not been formally assessed and cannot be excluded: this should be examined quantitatively in a future systematic review with meta-analysis.

## Conclusions

Noninvasive assessment of the AnT in endurance athletes is feasible and, for several methods, sufficiently accurate for practical use. HRV (DFA α1), NIRS and CP or CS currently show the most promising evidence to date, although device dependence, protocol standardization and individual-level limits of agreement remain important constraints on their interchangeable use with criterion methods; the heart rate deflection point and the talk test remain useful but coarser tools. No single method is optimal for every context; selection should be matched to the threshold of interest, the sport and the required precision, and reporting of device and protocol should be standardized. Definitive synthesis and, where feasible, meta-analysis will further quantify comparative accuracy.

## Data Availability

No new data were generated or analysed in support of this review.
